# Role of freeze-thaw cycles and chlorpyrifos insecticide use on diffuse Cd loss and sediment accumulation

**DOI:** 10.1038/srep27302

**Published:** 2016-06-02

**Authors:** Fangli Wang, Wei Ouyang, Fanghua Hao, Wei Jiao, Yushu Shan, Chunye Lin

**Affiliations:** 1 School of Environment, State Key Laboratory of Water Environment Simulation, Beijing Normal University, Beijing, 100875, China

## Abstract

Freeze-thaw cycles are predicted to increase in cold temperate regions. The potential influence of the interactions of freeze-thaw cycles and agrochemicals on the release of Cd into river water is unknown. In this study, the interactions of freeze-thaw cycles and chlorpyrifos (FC) on Cd mobility in soils were analysed. The spatial variability of soil Cd under long-term intensive tillage in a freeze-thaw agro-system was also identified. The temporal variation of sediment Cd was detected based on analysis of the sediment geochemistry. The results showed that FC increased soil Cd mobility, with an increase of approximately 10% in CaCl_2_-extractable Cd. The increased mobile fractions of water-soluble and exchangeable Cd originated from the decreased fraction of Fe-Mn-oxide-associated Cd and organic matter-bound Cd. The total Cd content in the surface soil followed the zonally decreasing trend of dry land > paddy land > natural land. The Cd concentrations and sedimentation rates of the sediment core generally increased from 1943 to 2013 due to agricultural exploration and farmland irrigation system construction, indicating an increase of the Cd input flux into water. The results provide valuable information about the soil Cd transport response to the influence of climatic and anthropogenic factors in cold intensive agro-systems.

Cadmium (Cd) is a highly mobile and toxic element that is notorious for its deleterious effects on the environment and human health^1^. Cd levels in the environment have increased several-fold in many parts of the world due to its increasingly higher input flux^2^,^3^. Anthropogenic activities have introduced greater inputs of Cd into soil than natural processes in recent decades^4^. Agricultural activities in arable soil (e.g., repeated sewage irrigation, spraying and fertilization) induce Cd losses due to overland flow and soil erosion, with Cd subsequently reaching aquatic environments^5^,^6^,^7^. Soil diffuse Cd loss has aggravated anthropogenic impacts on watershed environments for the last 20 years^8^. Therefore, agricultural activities are significant diffuse sources of Cd in the environment.

Long-term intensive agriculture in particular has been reported to account for the rapid deterioration of aquatic environments^8^,^9^. The consequences are strongly associated with the distribution and mobility of soil Cd. Land use conversions during long-term agricultural intensification directly/indirectly affect the geochemical position of Cd bound to the soil^10^. Intensive agricultural activities can significantly decrease/increase Cd levels in soils not only by contributing to soil Cd accumulation but also by directly impacting the soil physico-chemical properties through frequent ploughing and irrigation and the long-term application of agro-chemicals^4^. The co-existence of Cd and pesticides is very common in intensive agricultural zones relying on the application of high amounts of agro-chemicals^7^,^11^. Pesticides can compete with Cd for the same binding sites via electrostatic interactions and hydrogen bonding^12^. Moreover, pesticides have a notable impact on soil physico-chemical and microbial properties, such as soil pH, organic matter content and microbial activities^13^. These properties are key factors controlling soil Cd mobility^14^ and vary with different land uses and pesticide levels^13^,^15^. Thus, concerns about the impact of pesticides need to be addressed to better understand diffuse Cd losses under long-term tillage.

Moreover, surface and near-surface soils in cold climate regions experience freeze-thaw (FT) conditions in the winter. FT readily induces greater soil erosion and surface runoff^16^,^17^, which are important vectors for the transport of particulate Cd from soils to the aquatic environment^5^,^18^. FT also plays an important role in controlling soil properties. FT accelerates the release of Fe-Mn oxides and dissolved organic matter (DOM) in soil by destroying soil aggregates^19^,^20^, potentially promoting the formation of soluble complexing Cd^21^. Simultaneously, soil pH, a key factor controlling Cd mobility, correspondingly increases or decreases with the changing number of soil sorption sites of H^+^ on the surface of DOM^20^. Moreover, soil humidity and temperature during the FT process can significantly affect soil activities^22^, which strongly relate to Cd mobility^23^, raising the question of how soil Cd losses respond when FT interacts with pesticides. Although there are some reports on soil Cd in regions with intensive agriculture that also undergo FT, most of these studies were focused on the distribution and source identification^11^. Previous research that can answer this question is sparse, despite the verification that the mobile fractions of soil Cd are significantly increased by the interactions of FT and pesticides^20^. The response difference in soil Cd mobility is in need of further analysis.

Cd enrichment is observed in sediment due to the affinity of Cd for settling sediment particles in river water systems^24^. The sedimentation processes of sediment particles usually occur during the subsequent movement of Cd from various matrices to the aquatic environment^5^,^18^. The distribution of the Cd content in the river sediment core has been established to investigate long-term diffuse pollution, offering views of the natural and anthropogenic impacts at different spatial scales^9^. Therefore, the analysis of river sediment cores has been widely applied to track the changes in Cd accumulation through time via archiving past environmental conditions in aquatic environments^3^,^9^ to better explore the long-term diffuse pollution based on the illation of the geochemical history of a source region.

Based on the aforementioned background, this study aimed to (1) assess the spatial variability of soil Cd from 30-year land use conversions in a freeze-thaw intensive agricultural zone; (2) provide an innovative understanding of the loss dynamics of soil Cd responses to the interactions of FT and pesticides, highlighting the influence of climatic and anthropogenic factors; and (3) verify the two inferences based on analysing the temporal distribution of the Cd content in sediment particles and the ^210^Pb-based chronology of a sediment core.

## Results

### Spatial variation of soil Cd under long-term tillage

**Figure 1:**
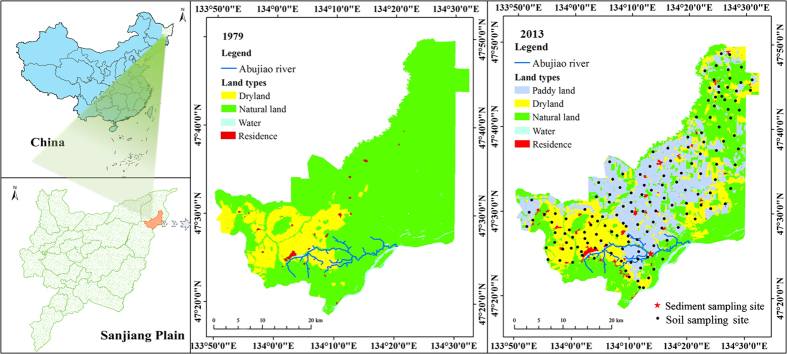
Location, land uses and sampling sites of the study area (using ArcGIS V 9.2 software, ESRI: Redlands, CA, URL: http://www.esri.com/software/arcgis).

**Figure 2:**
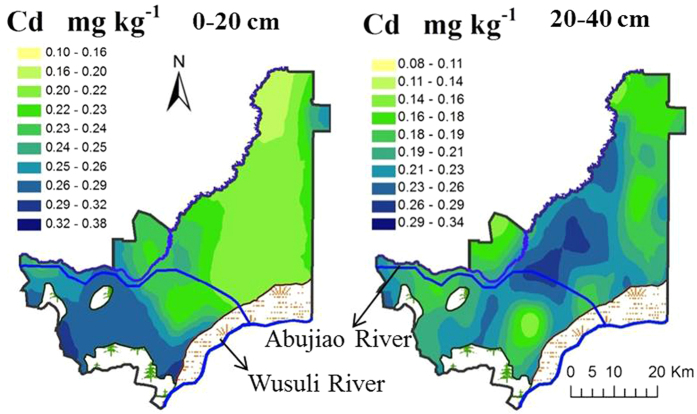
Spatial variation of the total Cd in arable soil (using ArcGIS V 9.2 software, ESRI: Redlands, CA, URL: http://www.esri.com/software/arcgis).

The two-term land uses here were categorized into five types: paddy land, dryland, natural land, water area and residence (Fig. 1). Over the three-decade period, 17.5% and 30.4% of natural land was converted to dryland and paddy land, respectively. Additionally, 22.5% of dryland was converted into paddy land. As exhibited in Fig. 2, there was a zonal and southwest-to-northeast decreasing trend of total Cd in the surface soil throughout the study area but a gradually increasing trend in the northeastern raised area. When coupling the spatial distribution of soil Cd with land uses, total Cd in the surface soil showed an overall tendency of dryland > paddy land > natural land.

The total Cd concentrations generally showed a much more regular distribution in the surface soil than in the subsoil, with ranges of 0.08–0.38 mg/kg and 0.10–0.34 mg/kg, respectively. According to the paired-samples T test results, the average content of total Cd in the surface soil of 0.24 ± 0.05 mg/kg generally was significantly (*P* < 0.05) higher than that in the subsoil, 0.21 ± 0.05 mg/kg. However, the total Cd concentrations in the middle part of the study area, where the main landscape was paddy land, were higher in the subsoil (0.27 ± 0.06 mg/kg) than in the surface soil (0.21 ± 0.04 mg/kg).

This tendency indicated higher Cd accumulation in arable soil and greater Cd loss in paddy land because the arable layer was mainly in the surface soil. Pesticide applications varied due to the different crop patterns, particularly in dryland and paddy land. Therefore, accounting for climatic factors, further analysis was implemented in the next section, focusing on the response differences of soil Cd mobility in arable soil to the interactions of FT and chlorpyrifos (CP) insecticide.

### Cd mobility in the arable soil response to the interactions of FT and CP

Table 1 illustrates the impact of FT and CP on soil Cd mobility in arable soil. In soils without CP, the values of *C-Cd*, *A-Cd*, *E-Cd* and *M-Cd* were in the range of 1.20–1.61, 1.39–1.84, 3.35–4.12 mg/kg and 21.6–23.4%, respectively. Generally, *C-Cd* and *E-Cd* gradually increased with increasing FT frequency, whereas *A-Cd* and *M-Cd* showed fluctuating tendencies. At the end of the nine FT cycles, all of the indexes of Cd mobility presented in this study increased. Specifically, *C-Cd* and *E-Cd* significantly increased by almost 27.2% and 22.9%, respectively. Either *C-Cd* or *E-Cd* at F9 was significantly higher than at other FT frequencies. Significant differences were observed between any two groups of *C-Cd*, despite the non-significant difference between F1 and the control. The maximum *A-Cd* concentration was at F1, which was significantly higher than at other FT frequencies, except F6. A significant increase in *A-Cd* was also observed at F6 compared with F1 and F3. For *M-Cd*, the sole significant difference was between the control and F3, where the maximum was observed.

As shown in Table 1, in soils with CP, the concentrations of *C-Cd, A-Cd*, *E-Cd* and *M-Cd* were in the ranges of 1.06–1.45, 2.75–2.26, 3.24–3.93 mg/kg and 24.0–30.2%, respectively. Generally, the fluctuating and increasing tendencies of the Cd mobility (except *A-Cd*) in soils with CP were generally similar to the cases in soils without CP. However, as a function of CP, the increment of *C-Cd*, *E-Cd* and *M-Cd* generated from FT was decreased by 10.9%, 56.3%, 19.5%. The concentrations of *E-Cd* and *A-Cd* were significantly increased by 9.76% and decreased by 27.5% at the end of the nine FT cycles, respectively. There were no significant differences in *C-Cd* or *M-Cd* among the various treatments. A significant difference in *A-Cd* was found between any two groups, except for the pair of P3–P6. The *E-Cd* concentrations related to P6 and P9 were notably higher than at the other frequencies. CP induced a significant decrease in the *C-Cd* concentration by 16.2 ± 4.54%, whereas the *M-Cd* value significantly increased by 7.45 ± 2.90%. However, no significant variation of *A-Cd* or *E-Cd* from CP addition was observed. Different changes in Cd mobility indicated interactive effects of FT and CP on the potential loss of soil Cd. To further analyse how these changes were produced, the interactions of FT and CP on soil Cd fractionation were investigated; this is described in the next section.

### Cd fractionation in the arable soil response to the combined impact of FT and CP

**Figure 3:**
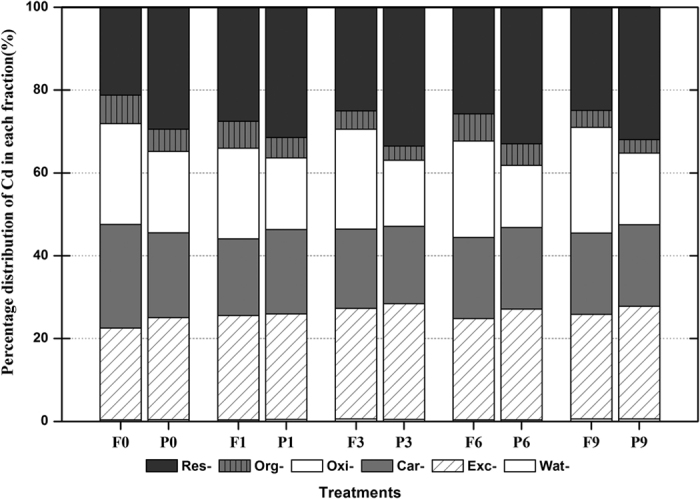
Changes in the Cd fractionation in arable soil resulting from the impact of freeze-thaw cycling (FT) and chlorpyrifos (CP).

The changes in Cd fractionation in arable soil resulting from the impact of FT and CP are illustrated in Fig. 3. Six fractions were obtained: water-soluble Cd (*Wat-Cd*), exchangeable Cd (*Exc-Cd*), carbonate-bound Cd (*Car-Cd*), Fe-Mn-oxide-associated Cd (*Oxi-Cd*), organic matter-bound Cd (*Org-Cd*) and residual Cd (*Res-Cd*). In soils without and with CP, the six Cd fractions fluctuated with increasing FT frequency. Generally, in soils without CP, the increased content of *Wat-Cd* (56.0%) and *Exc-Cd* (14.0%) resulting from FT mainly originated from the decreased *Org-Cd* content (40.3%) at the end of the nine FT cycles. Once CP was added to the soils, the increased content of *Wat-Cd* (32.5%), *Exc-Cd* (10.5%) and *Res-Cd* (8.56%) resulting from FT was caused by the decreased *Oxi-Cd* content (12.1%) and the decreased *Org-Cd* content (39.4%). In soils with CP, CP significantly weakened the changes caused by FT in the soil Cd factions. Moreover, CP significantly increased the content of *Wat-Cd*, *Exc-Cd* and *Res-Cd* by 55.8 ± 6.39%, 6.70 ± 0.93% and 23.6 ± 3.49%, respectively, whereas CP significantly decreased the content of *Oxi-Cd* and *Org-Cd* by 28.4 ± 3.70% and 21.8 ± 1.47%, respectively.

### Temporal distribution of Cd in the river sediment core

**Figure 4:**
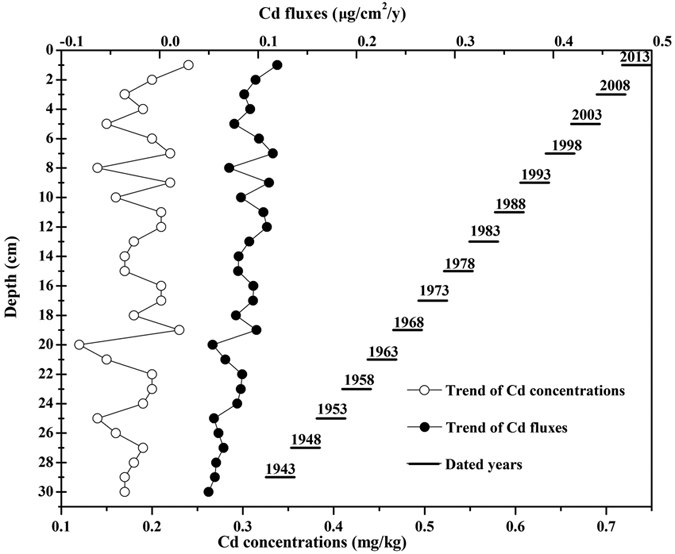
Cd distribution and ^210^Pb chronology of the sediment core from the outlet area.

To verify whether long-term intensive tillage and freeze-thaw conditions increase Cd flux from arable soil into the aquatic environment, the distributions of Cd and ^210^Pb_ex_ in a river sediment core from a watershed outlet were analysed and are shown in Table 2 and Fig. 4. The average mass sedimentation rate for the sediment core was 491 mg/cm^2^/y based on the results of the constant rate of supply (CRS) ^210^Pb-based model. The total Cd concentrations ranged from 0.14 to 0.24 mg/kg, and the maximum was in the surface layer. Then, the Cd sedimentation fluxes, which ranged from 0.05 to 0.12 μg/cm^2^/y, were computed by multiplying the mass sedimentation rates by the corresponding total Cd concentrations. The total concentrations, fluxes and sedimentation rates of Cd in the sediment core showed large fluctuations from 1943 to 2013, generally showing an increasing trend. The maximum sedimentation rate (523 mg/cm^2^/y) was observed in approximately 1998, with relatively larger total Cd concentration (0.22 mg/kg) and Cd flux (0.12 mg/cm^2^/y). The total concentration, flux and sedimentation rate of Cd in approximately 1984 were 0.21 mg/kg, 0.11 mg/cm^2^/y and 519 mg/cm^2^/y, respectively, relatively larger than in other years, except 1998.

## Discussion

### Spatial variation of soil Cd under long-term tillage

The total Cd concentrations in the surface soil of the study area exceeded the concentration in natural lands (0.10 ± 0.01 mg/kg, defined as the local background soil Cd). Some of the total Cd concentrations exceeded 0.30 mg/kg, which is the limitation (pH < 6.5) of Chinese soil standards for agricultural soils (GB 15618-1995). This phenomenon indicates an accumulating tendency of soil Cd, which is mainly due to the long-term high Cd input flux into soil from the use of pesticides, fertilizers and mulch, which strongly varies with different land use types and cultivation histories^11^,^25^,^26^. Moreover, runoff and soil erosion are also affected by the groundover structure, such as the type, the zoning and the density of the plants^27^. This factor also explains why the total soil Cd levels follow the trend of dryland > paddy land > natural land. Nevertheless, the mean value of total Cd concentration in the arable layer of this study (0.24 mg/kg) is much lower than in some industry-based areas at lower latitudes, such as Changsha (6.90 mg/kg), Hong Kong (0.62 mg/kg), and India (4.65 mg/kg)^28^. In addition to the fairly low inputs of soil Cd from atmospheric deposition, mining activities and municipal solid waste, there are other reasons for the spatial characteristics.

The study area locates in a large-scale intensive farm, which is different from small-scale farms. First, large changes in terms of land use usually occur in intensive agro-systems, which can effectively control CO_2_ emissions^15^,^29^. This process further affects carbonate buffering in the soil, which provides Cd carbonates or hydroxides as precipitates, complexes and secondary minerals that can lead to Cd solidification^14^. On this basis, the content of labile Cd in the soil is increased, resulting in an increase in the diffuse Cd loss from arable soil into rivers. Second, compacted soil caused by large agricultural machines usually has larger bulk density, fewer large pores and smaller infiltration rates, resulting in an increase in both runoff and soil erosion^27^. These events promote the loss of particulate Cd. Third, the organic matter (OM) content is much lower in the arable soil of the study area due to the greater rate of soil respiration compared to natural lands^25^. OM acts as a fundamental factor controlling Cd sorption by soils due to the large specific surface area and the elevated negative charge^4^. Thus, Cd complexes with OM in arable soils, which causes the loss of OM adsorbed-Cd with the loss of OM.

Moreover, in contrast to low-latitude regions, the impact of FT and agricultural intensification may play an important role in influencing soil Cd concentrations during the long, cold winters in the study area. Our previous research verified that the long-term intensive tillage in the study area resulted in a notable increase in surface field runoff^30^. Both FT and continuously frozen soil can generate greater soil particulate losses and greater runoff^16^,^17^. Therefore, more particulate Cd is removed, which contributes to the vast majority of field diffuse heavy metal loss^18^,^31^. Moreover, FT accelerates the process of carbon (C) and nitrogen (N) emissions (i.e., carbon dioxide, methane and nitrous oxide) under frequent tillage^19^,^26^. Such emissions are more significant due to the extremely fertile soil in this study area than in temperate regions. Based on this background, OM adsorbed-Cd is released into the soil liquid phase, enhancing the transport ability of soil Cd. This impact may be much weaker when considering the fact that the sorption-desorption process of Cd to OM is reversible. This reversibility is mainly dependent on soil pH, temperature, water condition, Eh values, cation exchangeable capacity and soil activity and their interactions^20^,^32^,^33^. However, this reversible sorption of soil Cd occurs rapidly (within 0.5 h), whereas the transfer of reversibly sorbed Cd into the irreversibly sorbed fractions is a significantly slower process^34^. Furthermore, microbial activities are suppressed in frozen soil^22^, resulting in the accumulation of organic matter (OM) in the deep humus horizon^25^. Cd bound to OM in the arable layer thereby migrates to deeper layers.

### Interactions of FT and CP on Cd mobility in arable soil

The results of the FT-CP experiments showed that *A-Cd*, *M-Cd* and all six Cd fractions fluctuated with the FT frequency, increasing in soils both with and without CP. One possible reason is due to the notable impact of FT on the soil pH^35^, which is highly related to the Cd mobility and most of the Cd fractions^14^. FT can alter the number of soil sorption sites of H^+^ based on destroyed soil aggregates or gathered fine^36^. Fe-Mn oxides are released, and dissolved OM is increased by destroying the soil aggregates^19^,^20^. On this basis, soil Cd mobility/solubility is increased by forming soluble complexes^23^. Therefore, the concentrations of *C-Cd* and *E-Cd* significantly increased at the end of the nine FT cycles in this study because the stable fractions of soil Cd (*Oxi-Cd* and *Org-Cd*) are converted into mobile factions (*Wat-Cd* and *Exc-Cd*) in this study. Cd is immobilized or mobilized by increased porosity and permeability due to the increased dissolved OM^21^,^36^. The increased OM can reduce the rate of reversibly sorbed Cd transfer into the irreversibly sorbed fractions, whereas the transfer rate increases with increasing temperature^34^. Cd desorption by DOM is easily promoted by FT due to the decreases in soil pH and the free iron oxides content^35^. Moreover, the impact of FT increases with the increasing FT frequency, i.e., acting time^20^. This process is strongly controlled by the temperature and the acting time^14^. Thus, the contents of *Exc-Cd*, *Oxi-Cd* and *Org-Cd* varied with different FT frequencies in this study, resulting in the fluctuating tendencies for *A-Cd* and *M-Cd*.

Moreover, the dissolved Cd^2+^ is transferred due to the migration of the unfrozen water to the frozen front during the FT process^27^,^37^. The increased temperature during the thawing process is beneficial for ion exchange sorption but detrimental to specific sorption^1^, which increases the concentrations of *Wat-Cd* and *Exc-Cd*, resulting in an increase in Cd mobility (*A-Cd*, *C-Cd*, *E-Cd* and *M-Cd*) at the end of the nine FT cycles. Furthermore, the microbial structure and functions of soil are sensitive to soil humidity and temperature^22^. The freezing process can cause a significant increase in the total amount of free amino acids and sugars, in combination with an increase in soil respiration and dehydrogenase activity^20^,^38^. In response, the soil organic matter (SOM) increases and the pH decreases. Cd is likely to bind with SOM under low soil pH conditions^14^. However, the increased pH-dependent cation exchange sites of OM can counteract this process via increasing the soil pH and cation exchange capacity. Thus, the *Oxi-Cd* concentration increased in this study at the end of the nine FT cycles in soils without CP, whereas the Org-Cd concentration significantly decreased.

However, the response of Cd mobility to FT can also be altered by CP via its ecological effects, organic effects and coordination reaction^13^. CP competes with Cd for sorption sites through electrostatic interactions and hydrogen bonding^39^. Additionally, CP significantly suppresses the activity of soil enzymes and reduces the buffering capacity via initial biotic transformation into chlorpyrifos-oxon^13^,^40^. This event causes further changes in soil SOM and pH, which are key factors controlling Cd mobility^14^. The disturbance of CP results in less sensitivity of Cd mobility to FT, which is demonstrated by the smaller increase of *C-Cd* (10.9%), *E-Cd* (56.3%) and *M-Cd* (19.5%) resulting from the interactions of FT and CP in this study. These results support the fact that natural factors (such as temperature, snowfall and other coexisting chemicals) not only modify the effects of chemicals but also indirectly influence them through their interactions^41^,^42^.

### Potential environmental hazard of Cd accumulation under long-term tillage

Once Cd has entered the soil, in addition to being retained in the soil, it may gradually transfer into rivers by the interactive effects of climatic and anthropogenic factors, such as precipitation (rainfall/snowfall), temperature and long-term tillage^5^,^27^,^30^. The lower total Cd concentrations of this study area in the arable layer compared with other low-latitude areas indicate that large Cd losses will easily occur under frequent intensive tillage. Moreover, the interactions of FT and CP significantly increased the Cd mobility directly via competing for the sorption sites and indirectly via altering the soil properties. Based on these results, the soil Cd losses result in larger input fluxes into the aquatic environment, which are strongly related to anthropogenic activities, field runoff and soil erosion^29^. During this process, diffuse heavy metal pollution occurs mainly in particulate form^9^,^31^. The deep humus horizon in the local soil is vulnerable to soil erosion as a function of FT^11^. Soil erosion has an affinity for the transportation of heavy metals that are bound strongly to soil colloids and OM, which is especially true for cultivated soil^31^. Thus, in addition to particulate Cd, parts of the exchangeable and OM-bounded Cd play a role in field diffuse Cd losses.

The sedimentation rate represents the variation in input flux at the watershed scale^43^. The general increases in the Cd concentration and sedimentation rate of the sediment core from 1970 to 2013 are mainly due to the increased intensive disturbance during the expanded agricultural exploration. This conclusion is supported by the fact that the sediment accumulated-Cd had an average enrichment factor (EF) of 1.56 and an anthropogenic contribution (The sediment accumulated-Cd resulting from agricultural activities, such as ploughing, agro-chemicals applications and irrigation.) of 34.5% in our previous study^44^, based on a nearby river in the Sanjiang Plain. This research indicates that sediment Cd accumulation has a strong relationship with anthropogenic factors (including land use changes and agro-chemicals applications). During this period, approximately 4927 m^2^ of natural land and 744 m^2^ of dryland were converted into paddy land at the scale of the river catchment due to the introduction of rice planting in the mid-1970s^25^. In addition to expanding the farmland, a catastrophic flood occurred in 1998, and large amounts of farmland irrigation systems were newly-built and enlarged in approximately 1984 to support farmland irrigation and water conservancy in the study area. Hydrological fluctuations (e.g., extensive runoff and erosion events) may occur during the agricultural irrigation process from year-to-year, enhancing soil particulate formation and transport^31^,^45^. These events potentially explain why the total concentrations, fluxes and sedimentation rates of Cd were relatively larger in approximately 1998 and in approximately 1984 than in other years.

The maximum Cd concentration at the surface layer indicates that a substantial increase in the watershed loading of Cd occurred in recent years^9^. The geographic location in the Chinese northeast border regions and the relatively lower industrialization of the study area greatly reduce the possibility of the Cd sources being derived from atmospheric deposition and industrial solid waste^11^. Therefore, it is speculated that Cd in the sediments mainly originates from intensive agricultural activities, which includes land use conversion, spraying and fertilization. This conclusion is consistent with previous studies^8^,^9^. FT readily enhances the impact of agricultural practices by increasing agricultural runoff and soil erosion as well as soil Cd mobility. This fact results in greater transfer of particulate Cd, as well as dissolved Cd and Cd complexes in arable soil, to river water.

These results indicate that frequent agricultural practices in freeze-thaw farmlands potentially increase the Cd input flux into the aquatic environment. The results provide valuable information about the soil Cd transport response to FT and long-term intensive tillage, highlighting the influence of climatic and anthropogenic factors in cold intensive agro-systems.

## Materials and Methods

### Study area description

The study area is located in the Sanjiang Plain of Northeast China (47°18′–47°50′N, 133°50′–134°33′E) (Fig. 1). As a core crop-production region of China, this area has undergone long-term intensive agricultural exploitation for more than 50 years^25^,^26^. The climate is continental with a mean annual temperature of 2.9 °C, ranging from −19.3 °C in January to 21.6 °C in July^20^. The area has an annual average freeze-thaw period of approximately 200 days and a maximal frozen depth of 141 cm^29^,^46^. The main soil type is Albic Luvisol (FAO, Food and Agriculture Organization), accounting for more than 95% of arable soil in the study area. The Albic Luvisol soil in this region has a 20 cm thickness of black soil layer. Due to the fertile and porous black soil on the surface and the impermeable clay horizon below, the soil in the study area is vulnerable to runoff and erosion, especially during the spring snowmelt and in the summer storm season^25^. The Abujiao River, which flows eastward, flows through the main types of land use (paddy land, dryland and natural land, Fig. 1) in the southern area. The total watershed area of the Abujiao River is approximately 142 km^2^, with an annual average water discharge of 1.11 m^3^/s. The hydrological function of the river is seasonal, with high water flows from May to September and a low water period between November and March of the next year.

### Sampling and total Cd analysis

To highlight the overall spatial variability of soil Cd from 30 years of agricultural intensification, topsoil (0–20 cm) and subsoil (20–40 cm) from 148 sites were randomly selected based on 1-km net grids in July 2013 (Fig. 1). Prior to sampling, plant residue was removed. At each sample site, five replicate samples were collected, homogenized by hand mixing, and then stored in plastic bags. Furthermore, a 30-cm-length river sediment core sample was collected from the watershed outlet of the farming area. The core sample was divided into 30 thin slices with an interval of 1 cm. All samples were air-dried, ground and sieved in plastic bags through a 100-mesh sieve. The subsamples were digested with a HF-HNO_3_-HClO_4_ acid mixture. The Cd concentrations in the digested solutions were determined by inductively coupled plasma optical emission spectrometry (ICP-OES, IRIS Intrepid II XSP, Thermo Electron, USA).

The sediment core was dated by the sedimentation rate, which was calculated using the CRS model due to the strong disturbance of anthropogenic activities^43^. The CRS model assumed a constant rate of the excess ^210^Pb (^210^Pb_ex_) from atmospheric fallout, allowing the concentration of sedimentary ^210^Pb_ex_ to vary inversely with the sediment accumulation rate. ^210^Pb_ex_ was computed by deducting the measured activity in secular equilibrium with ^226^Ra (^210^Pb_sup_, short for supported ^210^Pb) from the total activity of the isotope (^210^Pb_tot_)^47^. The ^210^Pb_tot_ activity was analysed by a coaxial-well-type, high-purity germanium detector (HPGe GWL series, ORTEC, USA) using its 46.5 keV gamma-ray emission. The ^226^Ra activity was obtained by its gamma-ray emission at 295.2 keV and 351.9 keV. The CRS model is more appropriate and reliable due to the better agreement with the ^137^Cs results and the catchment regulation^43^. The activities in this study were reported in Becquerel per kilogram (Bq/kg) of dry matter. The uncertainty associated with the two ^210^Pb activities (^210^Pb_tot_ and ^210^Pb_sup_) was less than 10%.

### Freeze-thaw and chlorpyrifos experiments

To investigate the interactions of climatic and anthropogenic factors on the loss of soil Cd, laboratory experiments were designed. The experiments were designed to address the interactive effects of FT and pesticides on Cd mobility, which can indicate the transport ability of Cd from the soil to the aquatic environment. CP was selected as the representative pesticide due to its wide application in controlling most agricultural pests^13^. To avoid too large initial error resulting from differences in the total soil Cd concentrations, the soil samples with the lowest total Cd levels (ranged from 0.08 to 0.09 mg/kg) were selected. The maximum water holding capacity was 41.2%, which was determined by the method in Muhammad *et al.*^48^.

One kilogram of soil and 10 mg L^−1^ of Cd^2+^ solution were added to 1 L of soil. Cd in soil was homogenized by incubating the mixture at 20 °C for approximately 2 months. The total content of Cd in soil was adjusted to 10 mg/kg. Then, the soil was air-dried, ground and sieved by a 2-mm mesh sieve. One gram of soil was weighed in plastic bags and adjusted to the maximum water holding capacity. The subsamples were stored in a dark incubator at 20 °C for 24 hours to reach equilibrium. Half of the subsamples were adjusted to 5 mg/kg CP. The subsamples with and without CP were subjected to the FT process with a frequency of 1, 3, 6 and 9. Subsamples subjected to the thawing temperature (20 °C) were used as the control group (0). The treated groups without and with CP were F (0, 1, 3, 6, 9) and P (0, 1, 3, 6, 9). Each treatment was performed with four parallel replicates. There were 40 groups and 160 samples in total. The temperature was set according to the local minimum and maximum monthly air temperature for several years, i.e., −19.3 °C and 21.6 °C. When the surface soil began to freeze, the air temperature was approximately 5–10 °C cooler than the soil temperature^49^. Thus, the freezing and thawing temperature were set to −10 °C and 20 °C, respectively, over a period of 24 hours for each, i.e., a FT cycle involved 2 days.

Three chemical methods were used to measure Cd mobility with 20 mL of 0.11 M HOAC (*A-Cd*), 25 mL of 0.05 M EDTA-2Na (*E-Cd*) and 20 mL of 0.01 M CaCl_2_ (*C-Cd*) extractions^14^. The volume of each reactant was selected based on its best performance in a previous study^50^,^51^. However, researchers do not agree on which reactant is the best to analyse soil Cd mobility due to the limitations in the reactants themselves. For example, CaCl_2_ solution only can extract water-soluble and exchangeable Cd, and HOAC solution has low reproducibility in soils with high carbonates levels. Therefore, three chemical methods were selected to make the soil Cd mobility analysis as accurate and comprehensive as possible. To interpret the changes in Cd mobility, Cd fractionations were performed using modified Tessier six-sequential extractions^52^. The content of each fraction of soil Cd was calculated as the ratio of each fraction to the sum of the extracted Cd in all six-sequential fractions. The relative index of Cd mobility (*M-Cd*) was calculated following Wang *et al.*^20^.

The total Cd concentrations in the extracts were determined by inductively coupled plasma optical emission spectrometry (ICP-OES, IRIS Intrepid II XSP, Thermo Electron, USA). Quality assurance and quality control procedures were implemented using duplicates, method blanks and standard reference materials (GBW07401). The average recovery of Cd was 98.4 ± 5.08%. The standard error of the duplicated samples was less than 5%. All chemicals were of analytical grade or better.

### Statistical analysis

Descriptive data and statistical analyses were performed using SPSS v.16.0 and Origin v.8.0, providing the means ± S.D. (standard deviation) of the four replicates. Statistically significant differences between two groups were identified by one-way analysis of variance (ANOVA) using Duncan’s test at a significance level of 0.05. Considering the superior linear unbiased estimator for quantities that vary in space^53^, the Kriging method was employed to predict the spatial distribution of the total Cd in unsampled soils using ArcGIS v.9.2. The type of the model and the parameters used in the Kriging method were reported in Table 3.

## Additional Information

**How to cite this article**: Wang, F. *et al.* Role of freeze-thaw cycles and chlorpyrifos insecticide use on diffuse Cd loss and sediment accumulation. *Sci. Rep.* **6**, 27302; doi: 10.1038/srep27302 (2016).

## Figures and Tables

**Table 1:** Impact of freeze-thaw cycling (FT) and chlorpyrifos (CP) on Cd mobility in arable soil.

FT frequency		*C-Cd* (mg/kg)				*A-Cd* (mg/kg)				*E-Cd* (mg/kg)				*M-Cd* (%)			
		CP0		CP1		CP0		CP1		CP0		CP1		CP0		CP1	
0	Mean	1.25	d	1.10	a	1.55	c	2.92	a	3.32	b	3.38	b	22.5	b	25.1	a
	SD	0.05		0.04		0.16		0.17		0.03		0.14		0.93		1.08	
1	Mean	1.32	d	1.13	ab	2.00	a	1.15	d	3.34	b	3.39	b	25.6	ab	26.9	a
	SD	0.02		0.01		0.19		0.14		0.03		0.18		2.07		1.73	
3	Mean	1.43	c	1.15	ab	1.50	c	1.90	c	3.39	b	3.43	b	27.3	a	28.4	a
	SD	0.07		0.01		0.12		0.12		0.08		0.03		1.81		2.27	
6	Mean	1.51	b	1.18	ab	1.86	ab	1.73	c	3.39	b	3.65	a	24.8	ab	27.1	a
	SD	0.07		0.09		0.14		0.14		0.37		0.03		2.62		2.39	
9	Mean	1.59	a	1.41	a	1.70	bc	2.12	b	4.09	a	3.71	a	25.9	ab	27.8	a
	SD	0.02		0.04		0.14		0.14		0.14		0.21		2.52		2.43	
		Increment			Reduction	Increment			Reduction	Increment			Reduction	Increment			Reduction
1		0.07		0.03	63.6%	0.45		−1.77	496%	0.02		0.01	58.3%	3.06		1.82	40.4%
3		0.18		0.05	72.8%	−0.05		−1.02	1954%	0.07		0.05	23.2%	4.82		3.31	31.4%
6		0.26		0.08	69.2%	0.31		−1.19	487%	0.07		0.27	−268%	2.35		2.04	13.2%
9		0.34		0.31	10.9%	0.15		−0.80	628%	0.77		0.33	56.3%	3.35		2.70	19.5%

Data in the same column with the same letter do not differ significantly at the 0.05 significance level; a > b > c > d.

**Table 2:** Total, supported and excess ^210^Pb concentrations in the sediment core and the CRS-modelled sedimentation rates.

Depth	^210^Pb_tot_	^210^Pb_sup_	^210^Pb_ex_	Mass sedimentation
(cm)	(Bq/kg)	(Bq/kg)	(Bq/kg)	rate (mg/cm^2^/y)
0–1	28.6 ± 2.7	14.4 ± 1.7	14.2 ± 1.0	498
1–2	28.6 ± 2.3	14.9 ± 2.4	13.7 ± 1.2	488
2–3	27.3 ± 1.5	14.8 ± 0.5	12.6 ± 1.1	505
3–4	26.4 ± 2.2	14.0 ± 1.8	12.4 ± 1.0	485
4–5	27.5 ± 1.6	16.3 ± 0.6	11.2 ± 1.1	506
5–6	25.7 ± 2.0	15.0 ± 1.2	10.7 ± 0.9	505
6–7	24.2 ± 2.3	14.4 ± 1.7	9.75 ± 0.6	523
7–8	24.3 ± 1.9	14.7 ± 1.3	9.58 ± 0.9	504
8–9	23.6 ± 2.1	14.5 ± 2.3	9.05 ± 1.0	505
9–10	21.7 ± 1.7	13.3 ± 1.1	8.39 ± 0.9	516
10–11	24.0 ± 1.9	15.8 ± 1.3	8.20 ± 0.7	502
11–12	21.2 ± 1.8	13.7 ± 1.5	7.52 ± 0.6	519
12–13	22.7 ± 2.1	15.4 ± 1.5	7.29 ± 0.6	506
13–14	21.5 ± 1.8	14.1 ± 1.2	7.40 ± 0.7	472
14–15	21.0 ± 1.8	14.0 ± 1.6	7.01 ± 0.4	470
15–16	23.2 ± 1.9	16.4 ± 1.5	6.78 ± 0.6	453
16–17	22.0 ± 1.6	15.6 ± 1.1	6.41 ± 0.6	453
17–18	22.7 ± 1.8	16.4 ± 1.3	6.30 ± 0.6	430
18–19	19.7 ± 1.6	13.8 ± 1.3	5.89 ± 0.5	428
19–20	20.3 ± 1.7	15.1 ± 1.4	5.18 ± 0.5	449
20–21	21.9 ± 1.9	17.0 ± 1.6	4.94 ± 0.4	445
21–22	21.0 ± 1.6	16.1 ± 1.3	4.91 ± 0.3	419
22–23	19.4 ± 1.4	14.8 ± 1.2	4.59 ± 0.4	413
23–24	20.5 ± 1.8	16.3 ± 1.5	4.19 ± 0.3	415
24–25	18.9 ± 1.2	14.8 ± 0.9	4.10 ± 0.4	395
25–26	20.1 ± 1.8	16.1 ± 1.6	4.01 ± 0.4	374
26–27	20.3 ± 1.0	16.3 ± 0.7	3.97 ± 0.3	343
27–28	18.6 ± 1.1	14.8 ± 0.9	3.80 ± 0.2	320
28–29	18.6 ± 0.7	15.3 ± 0.4	3.31 ± 0.3	330
29–30	19.0 ± 1.3	15.6 ± 1.0	3.41 ± 0.3	293

**Table 3:** Estimated parameter of the fitted variogram models in the Kriging method.

Layer	Nugget effect	Rang (km)	Nugget	Partial still	Model
0–20	30.5	16.4	648	2128	Exp
20–40	57.0	9.93	1464	3117	Exp
